# Design and assembly of a domestic water temperature, pH and turbidity monitoring system

**DOI:** 10.1186/s13104-021-05578-9

**Published:** 2021-04-30

**Authors:** Diana Rita Nanyanzi, Gilbert Gilibrays Ocen, Timothy Omara, Felix Bwire, Davis Matovu, Twaibu Semwogerere

**Affiliations:** 1 Department of Electrical and Computer Engineering, Faculty of Engineering, Busitema University, P.O. Box 236, Tororo, Uganda; 2 Department of Chemistry and Biochemistry, School of Sciences and Aerospace Studies, Moi University, Uasin Gishu County, P.O. Box 3900-30100, Eldoret, Kenya; 3 Africa Center of Excellence II in Phytochemicals, Textiles and Renewable Energy (ACE II PTRE), Moi University, Uasin Gishu County, P.O. Box 3900-30100, Eldoret, Kenya; 4 Department of Quality Control and Quality Assurance, AgroWays Uganda Limited, Plot 34-60, Kyabazinga Way, P.O. Box 1924, Jinja, Uganda

**Keywords:** Internet of Things, Water quality, Temperature, PH, Turbidity

## Abstract

**Objective:**

The aim of this study was to design a domestic water temperature, pH and turbidity monitoring system that could constantly log temperature, pH and turbidity of water and give alerts in case the parameters are outside the acceptable limits for potable water.

**Results:**

The system was designed, assembled and performed as expected. The study indicates that the proposed and designed system outperforms the existing manual monitoring system as it can constantly track and store changes in water quality. This could be used to prepare better treatment processes as well as identify problems in the water distribution system early enough.

**Supplementary Information:**

The online version contains supplementary material available at 10.1186/s13104-021-05578-9.

## Introduction

Availability of clean and safe water is a necessity for life on planet earth and the fuel for sustainable development [1–4]. However, declining water quality has become a global problem [5, 6]. Sub-Saharan Africa is one of the regions of the world with limited access to clean and safe water [7, 8]. Uganda is among the most hit countries in the Sub-Sahara with water scarcity problem [9]. Yearly, more than 1000 Ugandans die of water borne diseases [10]. The Ugandan government through Ministry of Environment and other private companies has tried to deliver safe water but only 71% of the urban population access piped water [11]. This implies that the rest of the population uses unsafe water which makes them highly susceptible to water-borne diseases like cholera, diarrhoea and typhoid.

Water distribution networks are designed with minimum levels of risk of contamination but factors like leakages, bursts, changes in temperature and under dosing of chlorine increases risk of contamination especially when they occur in vulnerable areas like along storm water channels or slums. This poses a dire need to monitor the changes in water quality along the distribution network. Water quality management relies on hazard identification through surveillance (routine and manual monitoring) [12]. To understand water quality and its probable impacts, there is need to collect and monitor data from time to time. With the industry 4.0 revolution [13], there is need to embrace new technologies to make water quality monitoring easy [14–18].

The current study was undertaken to develop a domestic water temperature, pH and turbidity monitoring system that can constantly monitor domestic water points like reservoirs and storage tanks, and automatically notify the concerned personnel when water quality falls below the set standards. This is in line with target 1 of the United Nations’ sixth Sustainable Development Goal which targets the achievement of “universal and equitable access to safe and affordable drinking water for all by 2030”.

## Main text

### Materials and system design

**Fig. 1:**
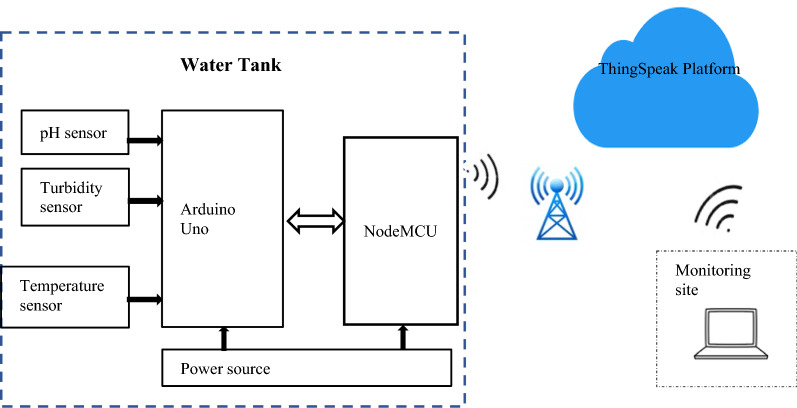
Block diagram of the domestic water temperature, pH and turbidity monitoring system

The proposed domestic water temperature, pH and turbidity monitoring system consisted of a network of 3 sensors for collecting data on temperature, pH and turbidity of water. These sensors were connected to an Arduino microcontroller which processes the data before relaying it to a cloud platform through a Wi-Fi module. From the cloud platform, the information is pulled and displayed on a website. The authorities in charge are then able to monitor the data for the different water quality parameters as well as analyse the data in form of graphs. Should the data collected vary from the set standards, then the authorities are alerted instantly. Figure 1 shows the block diagram of the proposed system.

The Arduino Uno used in this system is a microcontroller board based on ATmega 328P [17]. It was chosen because it has enough analog input pins to cater for the three analog sensors used. The waterproof DS18B20 temperature sensor is used to measure temperatures in wet environments with a simple 1-Wire interface [19]. It provides 9 to 12-bit Celsius temperature measurements and communicates over a 1-Wire bus that by definition requires only one data line (and ground) for communication with a central microprocessor [19]. The gravity analog pH sensor have a range of 0 to 14, which is measured between 0 to 60 °C [20]. The turbidity sensor detects suspended particles in water by measuring light transmittance and scattering rate which changes with the amount of total suspended solids [21]. It contains a light transmitter and receiver. When the water is clear, light scattering is minimal hence the light receiver receives the most amount of light. As turbidity of the water increases, the light receiver receives less light. The sensor detects when the light received is below a certain threshold.

The NodeMCU board was equipped with an ESP-12E module, which has an ESP8266 core processor with a module of 4 MB flash memory. With this board, the system was able to connect to internet and send the logged parameter data to the respective ThingSpeak channel fields. From the platform, the channel feeds are retrieved. That is, field data is displayed in the frontend application. Additional file 1: Figure S1 shows the algorithmic flow of the system when placed in water.

### Results and discussion

The current water quality monitoring tools range from traditional sample collection to remote sensing technologies in the world, with some time consuming, labour intensive or are too costly. In Uganda, routine monitoring is done by National Water and Sewerage Cooperation (NWSC) by sampling and testing once every quarter [11]. One of the key findings of monitoring drinking water samples by NWSC in areas of Kampala, Mukono and Wakiso districts was that delayed maintenance of the distribution network poses high risks of water contamination [22].

**Fig. 2:**
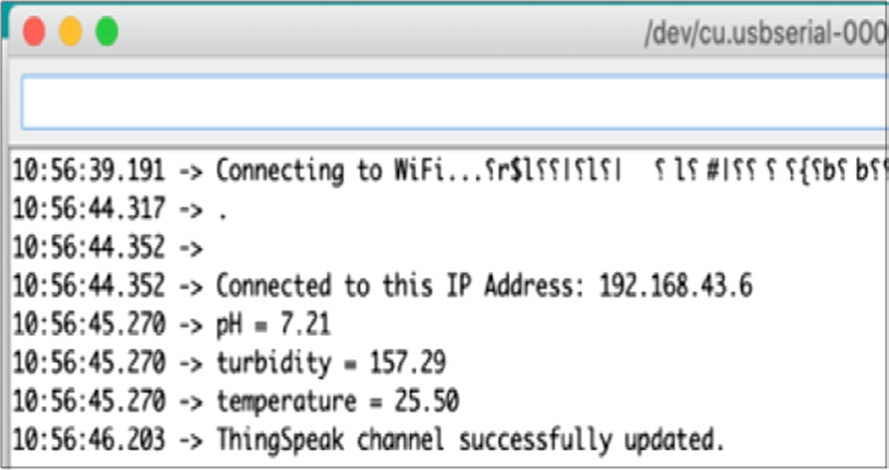
Connection of NodeMCU to ThingSpeak

**Fig. 3:**
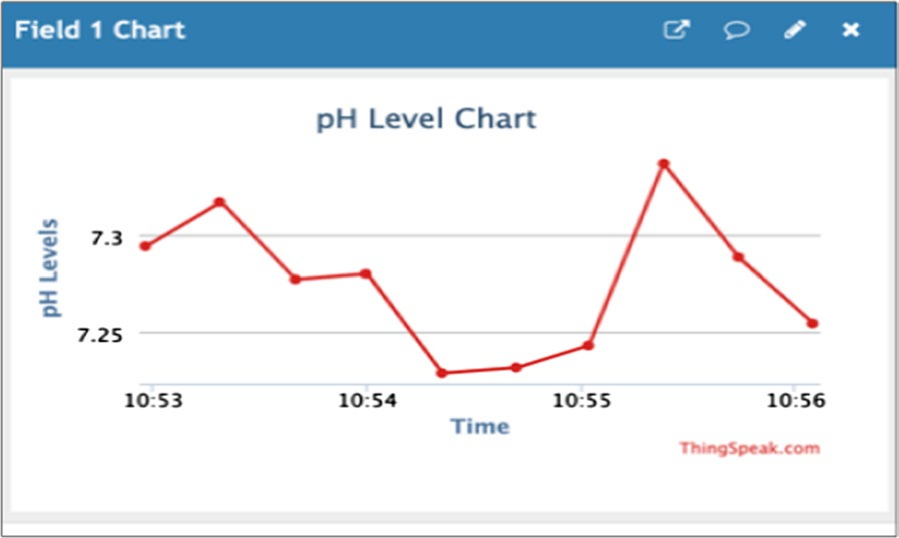
ThingSpeak pH chart

In this study, we designed a water quality monitoring system with sensors that are able to log data and process it with the Arduino Uno before it is sent serially to a NodeMCU. Figure 2 shows a successful connection of the NodeMCU to the internet. Once connected, the values sent serially from Arduino Uno board are pushed to the ThingSpeak platform for storage and analysis. On the ThingSpeak platform, the values for each parameter are recorded as they come in and are presented in graphical forms as shown in Fig. 3 and Additional file 2: Figure S2. These graphs are then displayed on the website.

When it was placed in clear tap water, the system indicated that the pH was 7.54 and turbidity was 0.00 which is in line with the NWSC standards for pumped and treated water. When placed in water with settled dirt, the system displayed that the turbidity was 2969 NTUs which makes the water unpalatable since it lies out of range of the acceptable standards (greater than 5 NTUs). When placed in muddy water, the system displayed that the turbidity was 3000 NTUs which means the water was unpalatable since its turbidity was beyond the acceptable limit. All the data were captured and displayed on the website as the most recent parameter status reading. The graphs and tabular format as presented in Additional file 3: Figure S3.

When temperature is below 20.0 °C, it indicates ‘‘LOW’’, greater than 26.0 °C indicates ‘‘HIGH’’ while ‘‘NORMAL’’ is for temperature between 20.0 and 25.5 °C. When the pH level is below 6.8, the status display on the website is “LOW”. When it is between 6.8–8.8, it shows “NORMAL” and “HIGH” when above 8.8. For turbidity values equal to or less than 5 NTUs, the status shown is “NORMAL” while for values above 5 NTUs, “HIGH” is displayed.

The designed system therefore had water quality sensors that transform detected chemical signals into electrical signals that can be translated into temperature, pH and turbidity measurements (Additional file 3: Figure S3). The results of this study corroborates a report by Azman et al. [23] who designed a low-cost nephelometric turbidity sensor for continual domestic water quality monitoring system. In another study [24], the authors reported that a sensor-based water pollution detection performed better than the traditional monitoring systems in homes and offices because it provided a real-time pH turbidity and temperature measurement which could enhance water quality monitoring. Another study [25] designed a system close to the one designed in the study but did not include the turbidity and temperature sensors. The current designed system is automatic, uses internet (Wi-Fi), is time saving, affordable with low maintenance cost and could prevent the spread of water-borne diseases through drinking water.

## Limitation

While the designed system saves on labour costs and time, it can only be used in a place with a good internet coverage to be able to relay the data to the cloud and consequently the web application. Further studies should incorporate more sensors for other water quality parameters such as nitrates, conductivity and total dissolved solids.

## Supplementary Information

**Additional file 1: Figure S1.** Logical flow of the domestic water temperature, pH and turbidity monitoring system.

**Additional file 2: Figure S2.** ThingSpeak turbidity chart.

**Additional file 3: Figure S3.** Website for visualization of the logged parameter data.

## Data Availability

The datasets supporting the conclusions of this study are available from the corresponding author upon reasonable request.
