# Supplementary material for: Design and assembly of a domestic water temperature, pH and turbidity monitoring system
Source: BMC Res Notes. 2021 Apr 30;14:161. doi: 10.1186/s13104-021-05578-9 (PMC8086135; doi:10.1186/s13104-021-05578-9)
Supplement: Supplementary file 1 — Additional file 1: Figure S1. Logical flow of the domestic water temperature, pH and turbidity monitoring system. [file 13104_2021_5578_MOESM1_ESM.docx]

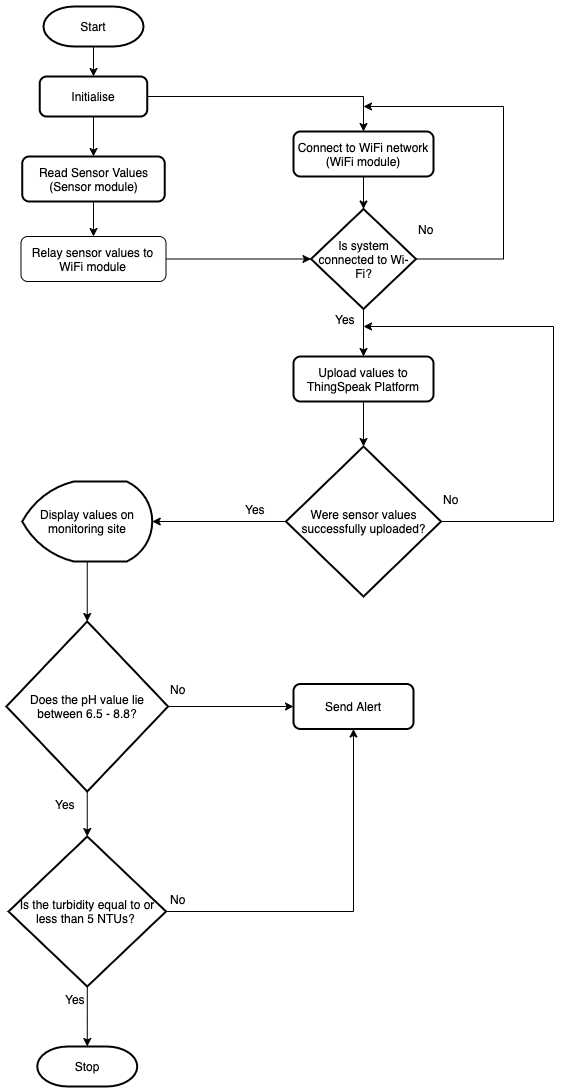


Additional File 1: Figure S1. Logical flow of the domestic water temperature, pH and turbidity monitoring system
