# Supplementary figures and images for: Design and assembly of a domestic water temperature, pH and turbidity monitoring system
Source: BMC Res Notes. 2021 Apr 30;14:161. doi: 10.1186/s13104-021-05578-9 (PMC8086135; doi:10.1186/s13104-021-05578-9)

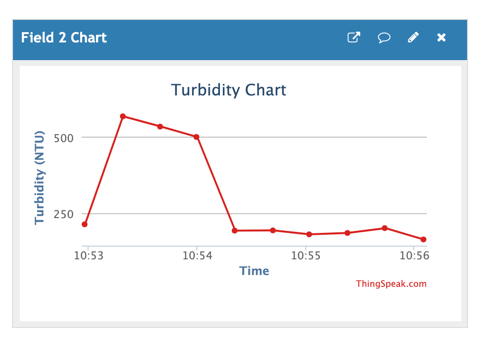


Additional File 2: Figure S2: ThingSpeak turbidity chart.

Supplement: Supplementary file 2 — Additional file 2: Figure S2. ThingSpeak turbidity chart. [file 13104_2021_5578_MOESM2_ESM.docx]

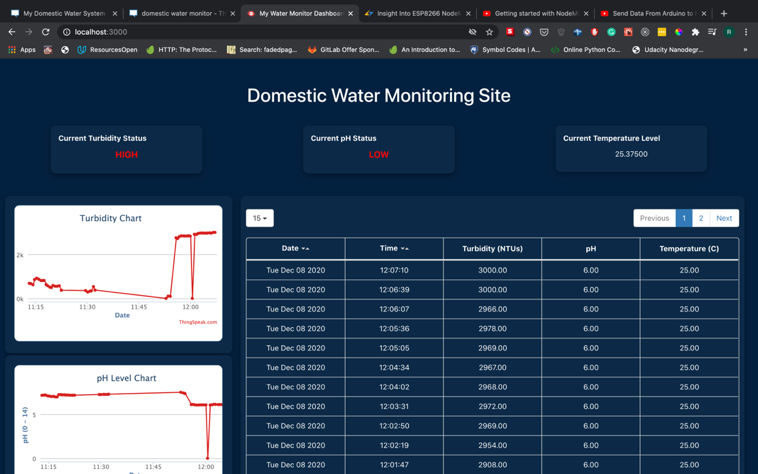


Additional File 3: Figure S3: Website for visualization of the logged parameter data.

Supplement: Supplementary file 3 — Additional file 3: Figure S3. Website for visualization of the logged parameter data. [file 13104_2021_5578_MOESM3_ESM.docx]
